# PLA2G16 promotes osteosarcoma metastasis and drug resistance via the MAPK pathway

**DOI:** 10.18632/oncotarget.7694

**Published:** 2016-02-25

**Authors:** Lin Li, Shoulei Liang, Amanda R. Wasylishen, Yanqin Zhang, Xueli Yang, Bingzheng Zhou, Luling Shan, Xiuxin Han, Tianyang Mu, Guowen Wang, Shunbin Xiong

**Affiliations:** ^1^ Institute of Cancer Stem Cell, Dalian Medical University Cancer Center, Dalian, China; ^2^ Department of Bone and Soft Tissue Tumors, Tianjin Medical University Cancer Institute and Hospital, National Clinical Research Center for Cancer, Key Laboratory of Cancer Prevention and Therapy, Tianjin, China; ^3^ Department of Genetics, The University of Texas, M.D. Anderson Cancer Center, Houston, Texas, United States of America

**Keywords:** PLA2G16, osteosarcoma, metastasis, drug sensitivity, MAPK

## Abstract

The prognosis of metastatic osteosarcoma is dismal and a better understanding of the mechanisms underlying disease progression is essential to improve treatment options and patient outcomes. We previously demonstrated Pla2g16 overexpression in mouse osteosarcoma contributes to metastasis phenotypes and increased expression of PLA2G16 is associated with metastasis and poor prognosis in human tumors. To further examine the mechanisms through which PLA2G16 contributes to human osteosarcoma metastasis and explore the potential of PLA2G16 as a therapeutic target in osteosarcoma, we generated a panel of human osteosarcoma cell lines expressing different levels of PLA2G16. The functional analyses of these cell lines demonstrated high levels of PLA2G16 expression increased osteosarcoma cell migration, invasion, clonogenic survival, and anchorage-independent colony formation. Importantly, this activity was dependent on the phospholipase activity of PLA2G16. Additionally, PLA2G16 overexpression decreased the sensitivity of cells to a panel of chemotherapeutic agents. Analysis of downstream pathways revealed the pro-metastasis functions of PLA2G16 were mediated through the MAPK pathway, as knockdown of PLA2G16 decreased ERK1/2 phosphorylation and pharmacological inhibition of MEK significantly repressed PLA2G16 mediated cell migration and clonogenic survival. Furthermore, PLA2G16 overexpression promoted xenograft tumor growth *in vivo*, and these tumors exhibit increased ERK1/2 phosphorylation. Lastly, the expression of PLA2G16 is strongly correlated with the increased ERK1/2 phosphorylation in human osteosarcoma samples, and the combined lesions are associated with reduced overall and metastasis-free survival. Collectively, these results demonstrate increased PLA2G16 expression activates the MAPK pathway to enhance osteosarcoma metastasis and may be a novel therapeutic target for these cancers.

## INTRODUCTION

Osteosarcoma is the most common primary malignant bone tumor in children, young adult and adolescents [1–3], and commonly arises in the femur (42%), the tibia (19%) and the humerus (10%) [4]. Despite newly-developed multi-agent chemotherapy and gradually improved surgical techniques, the overall survival rate since 1970s remains only approximately 60% [5]. Additionally, osteosarcoma is associated with a high propensity for lung metastasis and clinical analysis shows that a 5-year survival rate for patients with metastatic osteosarcoma is only 10% to 20% [3, 6]. Thus, it is highly desirable to identify novel biomarkers and therapeutic targets to help evaluate and treat osteosarcoma patients.

There is conflicting evidence in the literature about the functional contributions of PLA2G16 (also referred to as H-REV-107, HRASLS3 and AdPLA2) to tumorigenesis [7, 8]. PLA2G16 is reported to suppress *HRAS* induced transformation, and inhibit cell proliferation, colony formation, and promote apoptosis, and was considered as a tumor suppressor before its phospholipase activity was discovered [7, 9–11]. Interestingly, as a phospholipase, PLA2G16 generates lysophosphatidic acid (LPA) and free fatty acid (FFA) from phosphatidic acid [12, 13]. LPA is a critical signal transduction molecule and metabolism regulator that promotes tumor progression by modulating cytoskeletal changes, cell-cell contacts, cell survival, proliferation, invasion and metastasis through activating multiple signal pathways, such as HRAS, MAPK, RAC, RHO, PLC, AKT and Hippo-YAP pathways [14–20]. Moreover, FFAs including the arachidonic acid and other unsaturated fatty acids, which contributes to the production of prostaglandin Es, can also play an important role in cancer pathogenesis [12, 21]. *PLA2G16* null mice are resistant to high fat diet or leptin deficiency induced obesity through the PGE2-EP3-cAMP pathway [8], suggesting *PLA2G16* may contribute to tumor progression through altered metabolic pathways. Additionally, PLA2G16 is also reported to suppress protein phosphatase 2A (PP2A) activity in ovarian carcinoma cells [22]. Yet PP2A is a well-known tumor suppressor and often genetically mutated or inactivated in many leukemia and solid cancers [23–26]. Therefore PLA2G16 may have oncogenic roles in some human tumors. Moreover, high level of the PLA2G16 protein expression in the cytoplasm increased proliferation of a subset of non-small cell lung carcinomas, thus contributed to tumor progression and poor prognosis [27]. Notably, we previously demonstrated that expression of *Pla2g16* induced by mutant p53 in mouse osteosarcoma cells contributes to the increased metastatic features [28]. Importantly, PLA2G16 expression is associated with poor prognosis and metastasis in human osteosarcoma regardless of p53 status [29], which strongly supports that PLA2G16 play an important role in osteosarcoma progression and metastasis, yet the downstream pathways which mediate the oncogenic function of *PLA2G16* in human osteosarcoma remain unknown.

In addition, phospholipases are implicated in chemo resistance. Etoposide-induced cleavage of phospholipase C-γ1 represses apoptosis and contributes to chemo resistance in T leukaemia cells [30]. More closely, inhibition of phospholipase A_2_ activity leads to less apoptosis and chemo resistance in non-small cell lung cancer (NSCLC) [31]. The effect of PLA2G16 on drug sensitivity in human osteosarcoma remain unknown.

In this study, several human osteosarcoma cells with varying levels of PLA2G16 were used to assess the effect of *PLA2G16* expression on proliferation, clonogenic survival, anchorage-independent colony growth, invasion, migration and drug sensitivity. Additionally, Saos2 cells with PLA2G16 overexpression were injected subcutaneously in nude mice to determine the tumorigenic potential of PLA2G16 overexpressing cells. Furthermore, we also investigated the pathways downstream of *PLA2G16.* Our data reveal that the oncogenic activity of PLA2G16 is mediated in large part through the activation of the MAPK pathway. Thus, this study establishes *PLA2G16* as a therapeutic candidate for metastatic osteosarcoma in patients.

## RESULTS

### PLA2G16 promotes osteosarcoma cell proliferation, colony formation, migration and invasion

**Figure 1:**
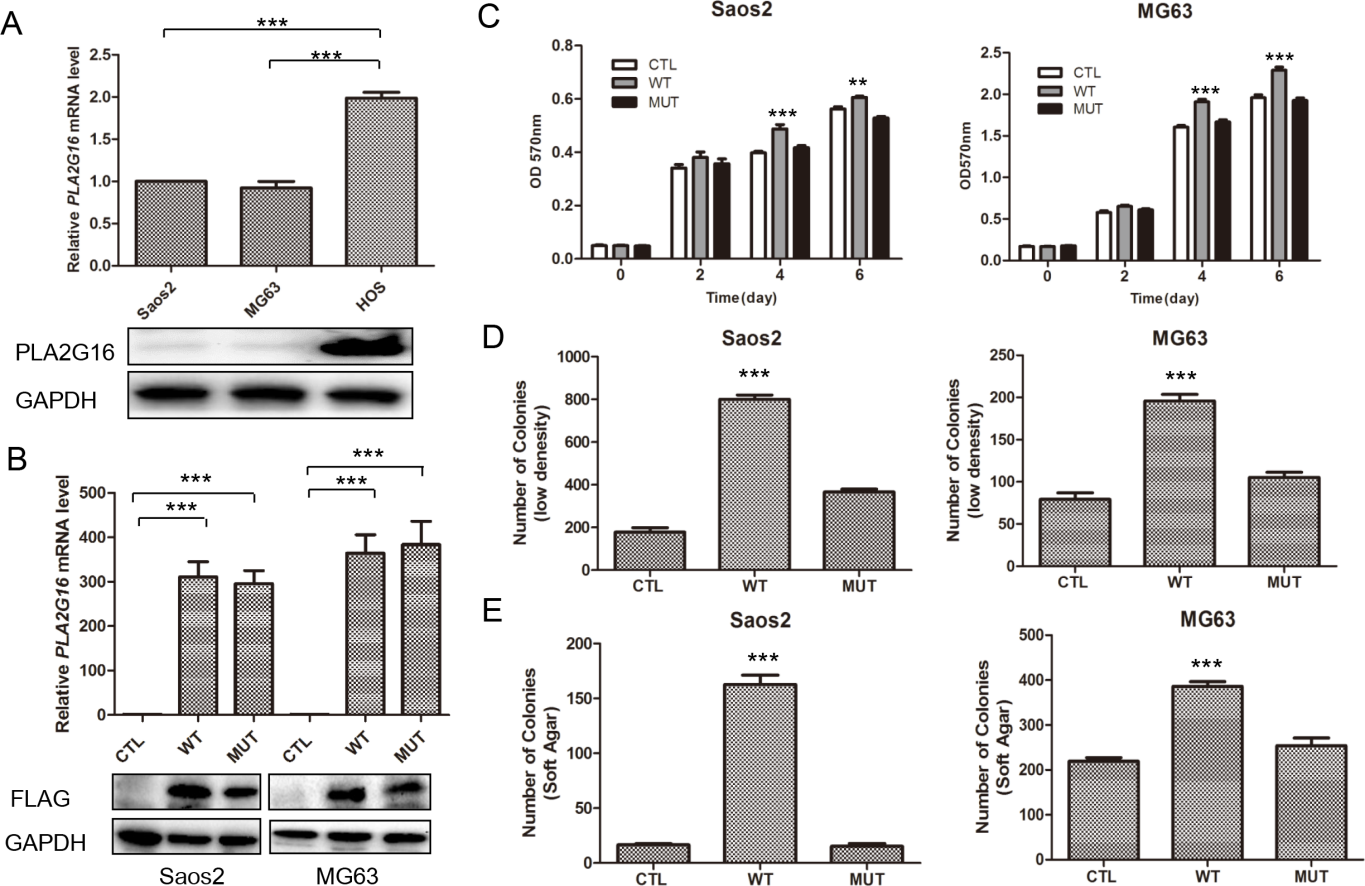
Overexpression of PLA2G16 increases osteosarcoma cell proliferation, clonogenicity and anchorage-independent colony formation (**A**) PLA2G16 expression levels in osteosarcoma cell lines as determined by qRT-PCR (upper) and western blotting analyses (lower). (**B**) *PLA2G16* or *PLA2G16-C113S* overexpression levels in Saos2 and MG63 cells as determined by qRT-PCR (upper) and western blot (lower) analyses. MTT analysis (**C**), Low density colony formation (**D**) and soft agar assays (**E**) were performed with PLA2G16 or PLA2G16-C113S overexpression cells. Results are shown as the mean ± SD of three independent experiments and one way ANOVA was used to determine the statistical significance between PLA2G16 overexpression (WT) and empty vector pBABE-puro (CTL) cells. ** indicates *p* < 0.01; *** indicates *p* < 0.001; CTL, pBABE-puro; WT, pBABE-FLAG-PLA2G16; MUT, pBABE-FLAG-PLA2G16-C113S.

**Figure 2:**
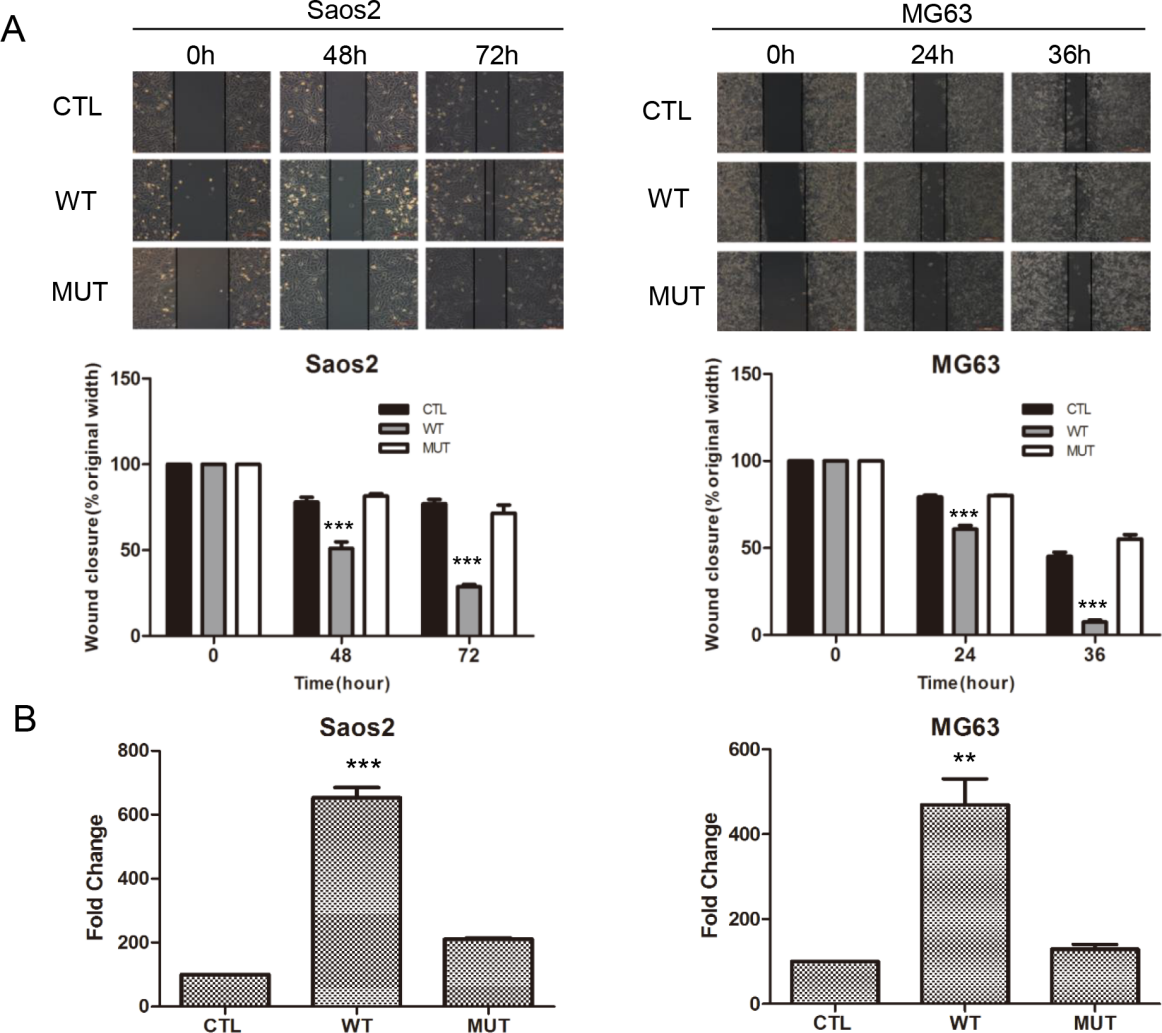
Overexpression of PLA2G16 enhances osteosarcoma cell migration and invasion (**A**) Wound-healing assay of PLA2G16 and PLA2G16-C113S overexpressed cells. All cancer cells were plated into 6-well plates with Ibidi culture inserts to form the wound and photos were taken every 12 hours by inverted microscope with a 10× magnification (upper). The average width of the wounds was determined by Image Proplus software (lower). (**B**) Cells invasion was determined by Matrigel transwell assays. Cells at the lower side of the membrane were photographed and counted. The graphic represent the mean and S.D. from three experiments and one way ANOVA was used to determine the statistical significance between PLA2G16 overexpression (WT) and empty vector pBABE-puro (CTL) cells. *** indicates *p* < 0.001;** indicates *p* < 0.01.

Our previous work indicated that *Pla2g16* can promote tumor progression and metastasis in mouse osteosarcoma cells [28]. Additionally, we demonstrated that increased PLA2G16 expression in osteosarcoma is associated with metastasis and poorer survival [29]. Thus, to further examine what metastatic properties can be induced by PLA2G16 overexpression and investigate the underlying mechanisms, we engineered overexpression and knockdown models of PLA2G16 in human osteosarcoma cell lines. We first examined the endogenous expression level of PLA2G16 in Saos2, MG63, and HOS cells. In comparison to Saos2 and MG63, HOS cells showed higher mRNA and protein expression of PLA2G16 by both the real time quantitative PCR and western blot analyses respectively (Figure 1A). We then generated Saos2 and MG63 cell lines with stable overexpression *PLA2G16* (WT) or *PLA2G16-C113* (MUT). The C113 mutation has a loss of lipase function mutation at amino acid 113 and is used to determine if the lipase activity is required [12, 13]. *PLA2G16* and *PLA2G16-C113* expression were significantly increased at both the mRNA and protein levels in Saos2 and MG63 cells (Figure 1B). MTT assays were performed to determine the effect of PLA2G16 overexpression to cell proliferation. These results indicate that *PLA2G16* overexpression significantly enhances cell proliferation in both cell lines when compared to both empty vector control (CTL) and the lipase-deficient mutant *PLA2G16-C113* (Figure 1C). Moreover, low-density colony formation assays clearly demonstrate that *PLA2G16* overexpression increases the clonogenic survival of cells (Figures 1D and S1A). In addition, we also carried out a soft agar colony formation assay to examine the effect of *PLA2G16* overexpression on anchorage-independent cell growth. Overexpression of *PLA2G16* caused a significant increase in both the number and size of the colonies in Saos2 and MG63 cells while overexpression of *PLA2G16-C113* had no effect (Figures 1E and S1B). Additionally cell migration and invasion were evaluated by wound-healing and Matrigel transwell assays, respectively. Saos2 and MG63 cells with *PLA2G16* overexpression showed a significant increase in migration (Figure 2A) and invasion (Figure 2B), while overexpressing of *PLA2G16-C113* had no effect (Figures 2A, 2B, S2). Notably, as the proliferation of *PLA2G16* overexpressing cells did not show significant difference during the first 48 hours (Figure 1C), and the cells were incubated with medium without FBS in the migration and invasion assays, it is unlikely that proliferation rates influenced migration and invasion. Combined these data strongly support that *PLA2G16* overexpression contributes to human osteosarcoma progression and metastasis, and this activity is dependent on the lipase activity.

**Figure 3:**
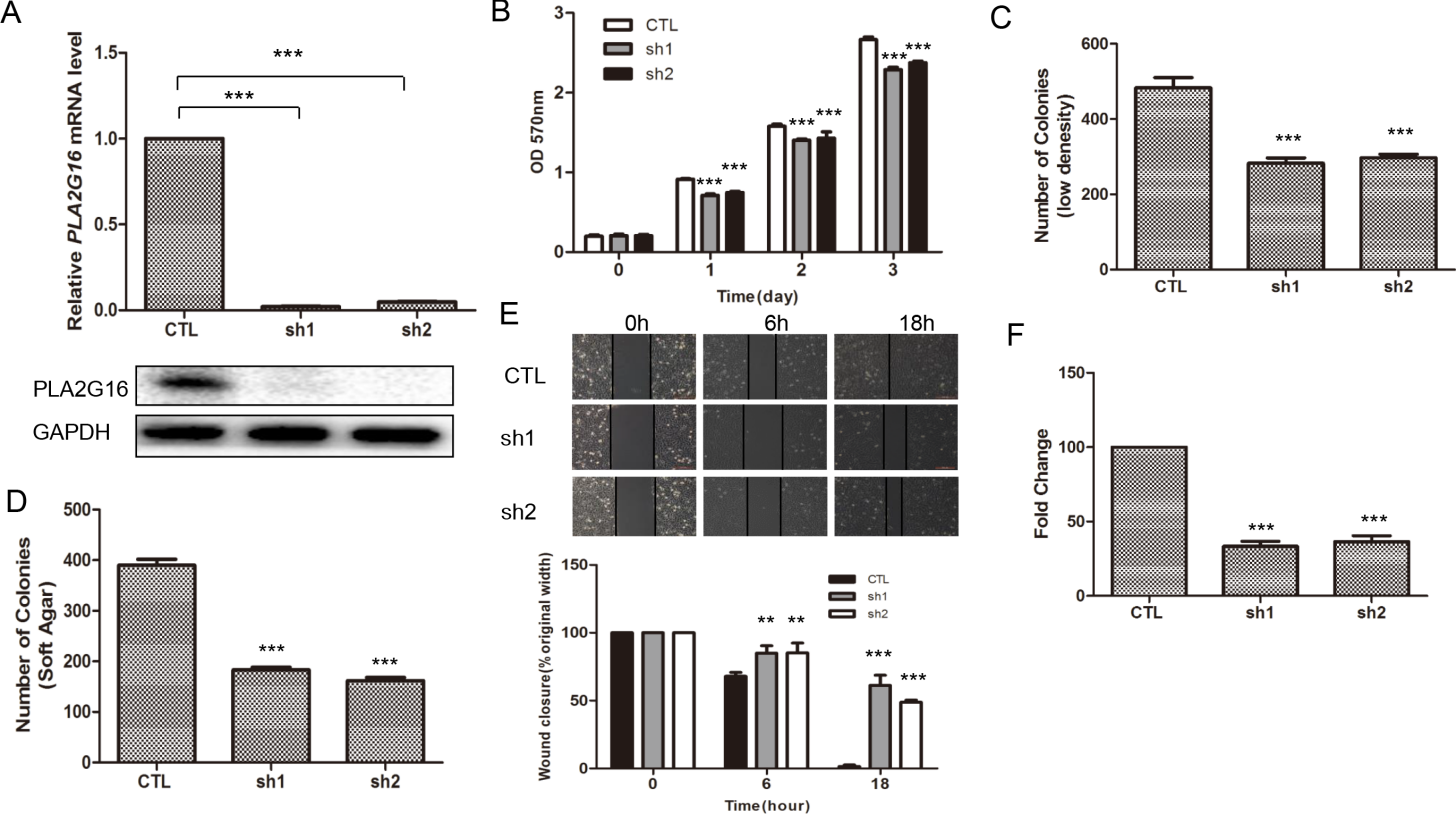
Knockdown of PLA2G16 decreases osteosarcoma cell proliferation, clonogenicity, anchorage-independent colony formation, migration and invasion (**A**) PLA2G16 expression in HOS cells with shRNA knockdown was determined by qRT-PCR (upper) and western blot (lower) analyses. MTT analyses (**B**), low density colony formation (**C**), Anchorage-independent growth (**D**), wound-healing (**E**) and Matrigel transwell (**F**) assays were performed in HOS cells with PLA2G16 knockdown. The colonies were counted from low density colony formation and soft agar assays after incubation and staining. Results are shown as the mean ± SD of three independent experiments and One way ANOVA was used for statistical analyses to compare sh1 or sh2 knockdown to control cells ** indicates *p* < 0.01; *** indicates *p* < 0.001; sh1 and sh2 were shRNAs against PLA2G16.

We previously demonstrated that knockdown of PLA2G16 in LM7 cells resulted in a clear reduction in migration and invasion [28]. LM7 are a subline of Saos2 cells that were enriched for their metastatic potential through tail vein injections in nude mice, and subsequently isolated from the tumor that formed in the lung. To further examine the role of *PLA2G16* in osteosarcoma in a more relevant human cell line, we knocked down *PLA2G16* expression in HOS cell line (Figure 1A). Similar to LM7 cells, two shRNAs were used to knock down *PLA2G16* in HOS cells, resulting in more than 90% reduction by both qRT-PCR and western blot analyses when compared to the EGFP knockdown control cells (Figure 3A). MTT analysis showed that knockdown of *PLA2G16* decreased HOS cell proliferation (Figure 3B). Clonogenic survival and soft agar colony formation were significantly repressed with *PLA2G16* knockdown (Figures 3C, 3D, S3A, and S3B). Interestingly, the colony size from knockdown cells were also smaller in soft agar experiments (Figure S3B). Similarly, *PLA2G16* knockdown in HOS cells resulted in decreased migration and invasion (Figures 3E, 3F and S3C). Therefore these data demonstrate that high levels of PLA2G16 expression increases multiple metastatic properties in osteosarcoma cells and it may be a novel therapeutic target for osteosarcoma.

### PLA2G16 reduces sensitivity to chemotherapy in osteosarcoma cells

**Figure 4:**
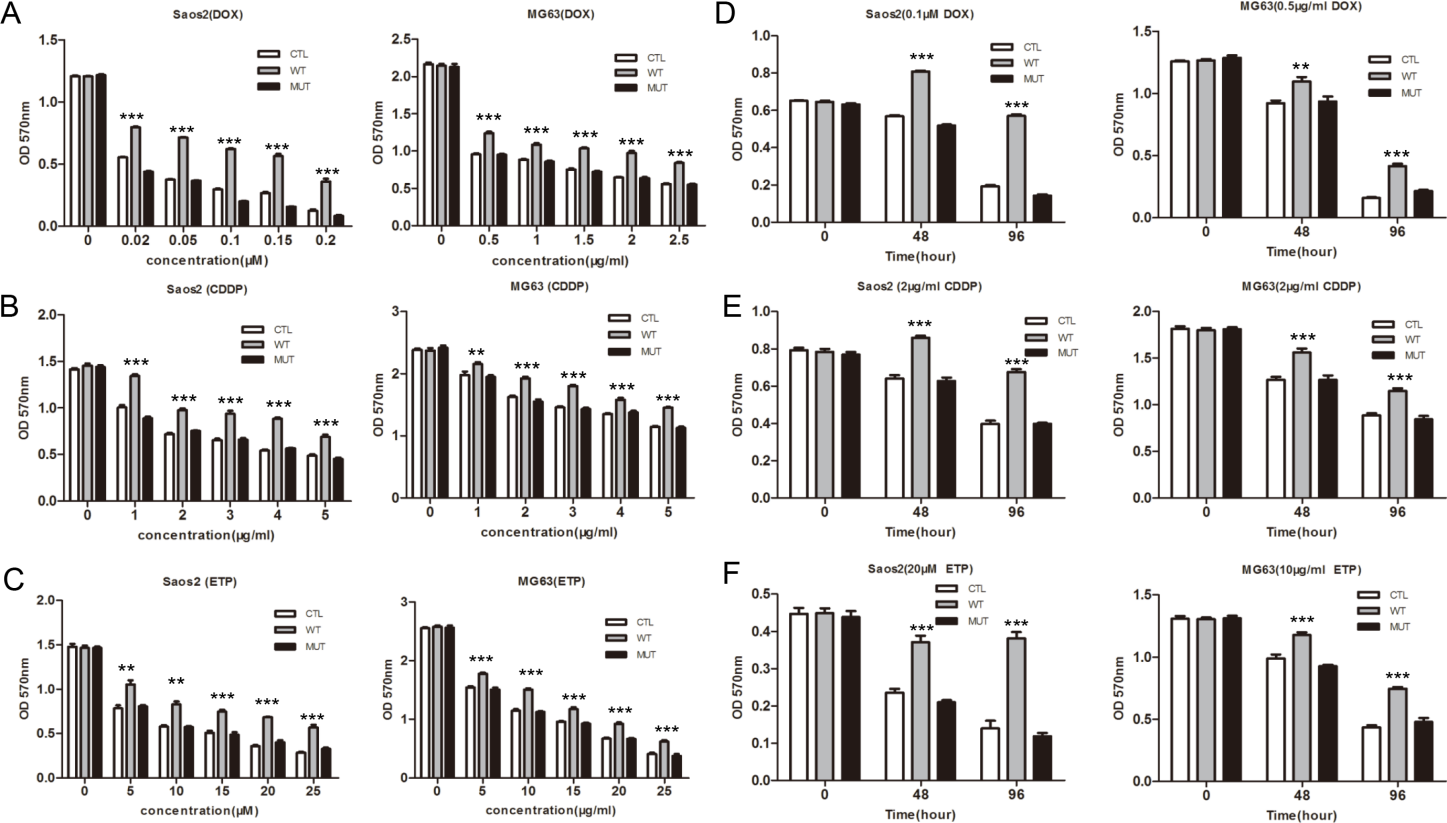
Overexpression of PLA2G16 decreases sensitivity to anticancer drugs in a dose- and time-dependent manner Cells were plated into 96-well plates and incubated with different concentration of doxorubicin (DOX) (**A**), cisplatin (CDDP) (**B**) and etoposide (ETP) (**C**) for 48 hours. Cells were incubated with DOX (**D**) or CDDP (**E**) and ETP (**F**) for different time period before the addition of MTT. The absorbance of 570 nm was measured after 4 hours with MTT. Results are shown as the mean ± SD of three independent experiments and One way ANOVA was used to determine the statistical significance between PLA2G16 overexpression (WT) and empty vector pBABE-puro (CTL) cells ** indicates *p* < 0.01; *** indicates *p* < 0.001.

**Figure 5:**
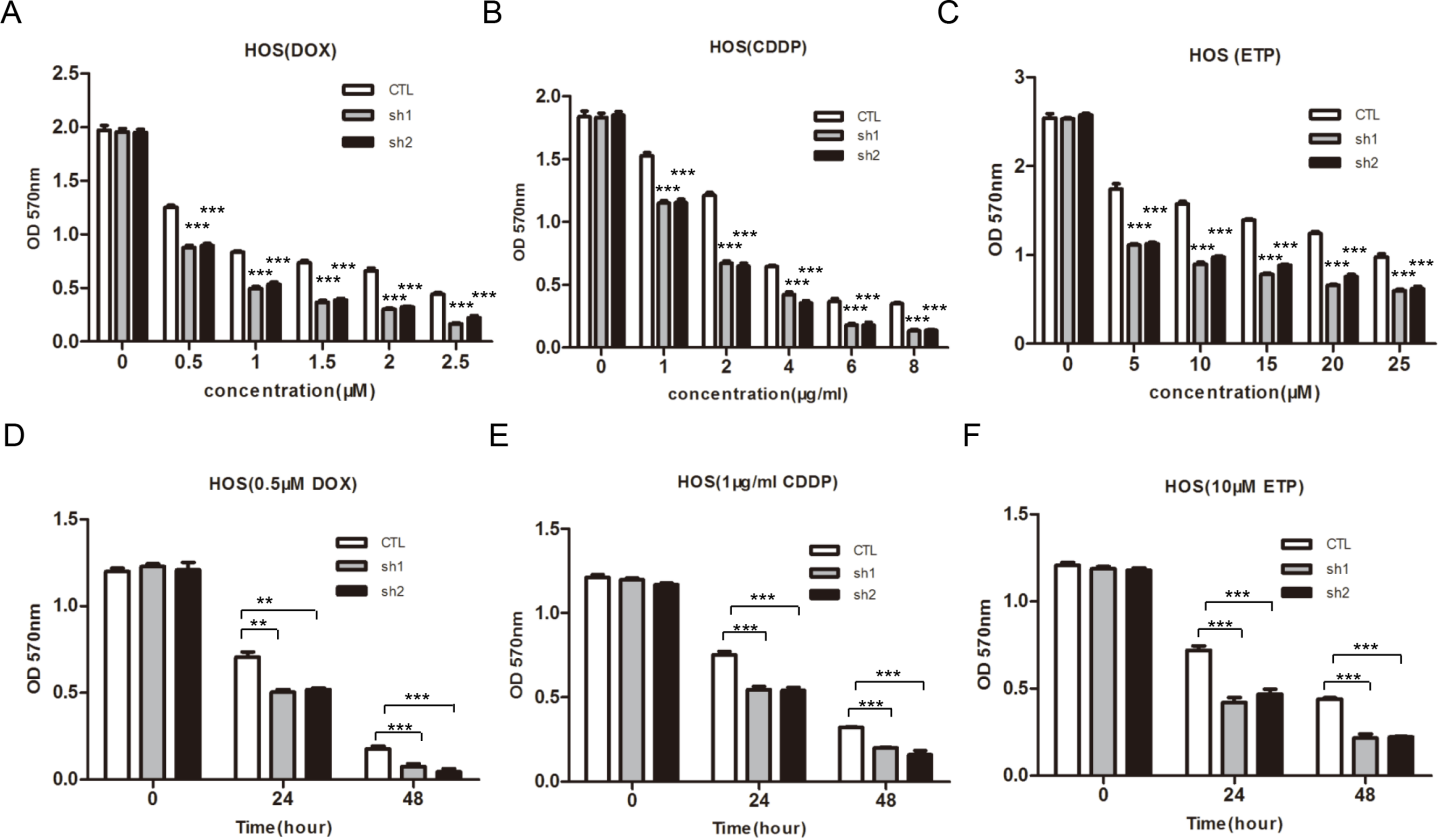
Knockdown of PLA2G16 enhances sensitivity to anticancer drugs in dose-and time-dependent manner in HOS cells (**A–F**) Cells were incubated with different concentration of DOX, CDDP and ETP for 48 hours or treated with DOX, CDDP and ETP for indicated time respectively before cells viability was determined by MTT assay. Results are shown as the mean ± SD of three independent experiments. One way ANOVA was used for statistical analyses to compare sh1 or sh2 knockdown to control cells ** indicates *p* < 0.01; *** indicates *p* < 0.001.

One of the reasons for the poor prognosis of osteosarcoma is the development of drug resistance. Since overexpression of PLA2G16 is implicated in cell survival and phospholipases are reported to increase chemo resistance, we next evaluated the effect of PLA2G16 overexpression on sensitivity to a panel of chemotherapeutics, including doxorubicin (DOX), cisplatin (CDDP) and etoposide (ETP). Saos2 and MG63 cells that stably expressing PLA2G16 (WT), PLA2G16-C113 (MUT) or the control vector (CTL) were incubated with different concentrations of DOX and CDDP for 48 hours respectively, and cell viability was determined by MTT assay. *PLA2G16* overexpressing cells were less sensitive to DOX, CDDP and ETP than CTL group and MUT group in a dose-dependent manner (Figure 4A, 4B and 4C). In addition, cells were also incubated with drugs for different times and cells overexpressing *PLA2G16* showed decreased sensitivity to DOX, CDDP and ETP in a time-dependent manner in both cell lines (Figure 4D, 4E and 4F). Similarly, we also assessed whether PLA2G16 knockdown could affect HOS cell drug sensitivity. The shRNA expressing HOS cells showed higher sensitivity to anticancer drug DOX, CDDP and ETP in a time- and dose-dependent manner (Figure 5A, 5B, 5C, 5D, 5E and 5F). Thus, the above results demonstrated that PLA2G16 expression may contribute to chemotherapy resistance, and it can be a potential target for the combination therapy using lipase inhibitors together with classic anticancer drugs in osteosarcoma patients.

### The oncogenic function of PLA2G16 is associated with cytoplasmic localization and is partially mediated through increased MAPK signaling in osteosarcoma cells

**Figure 6:**
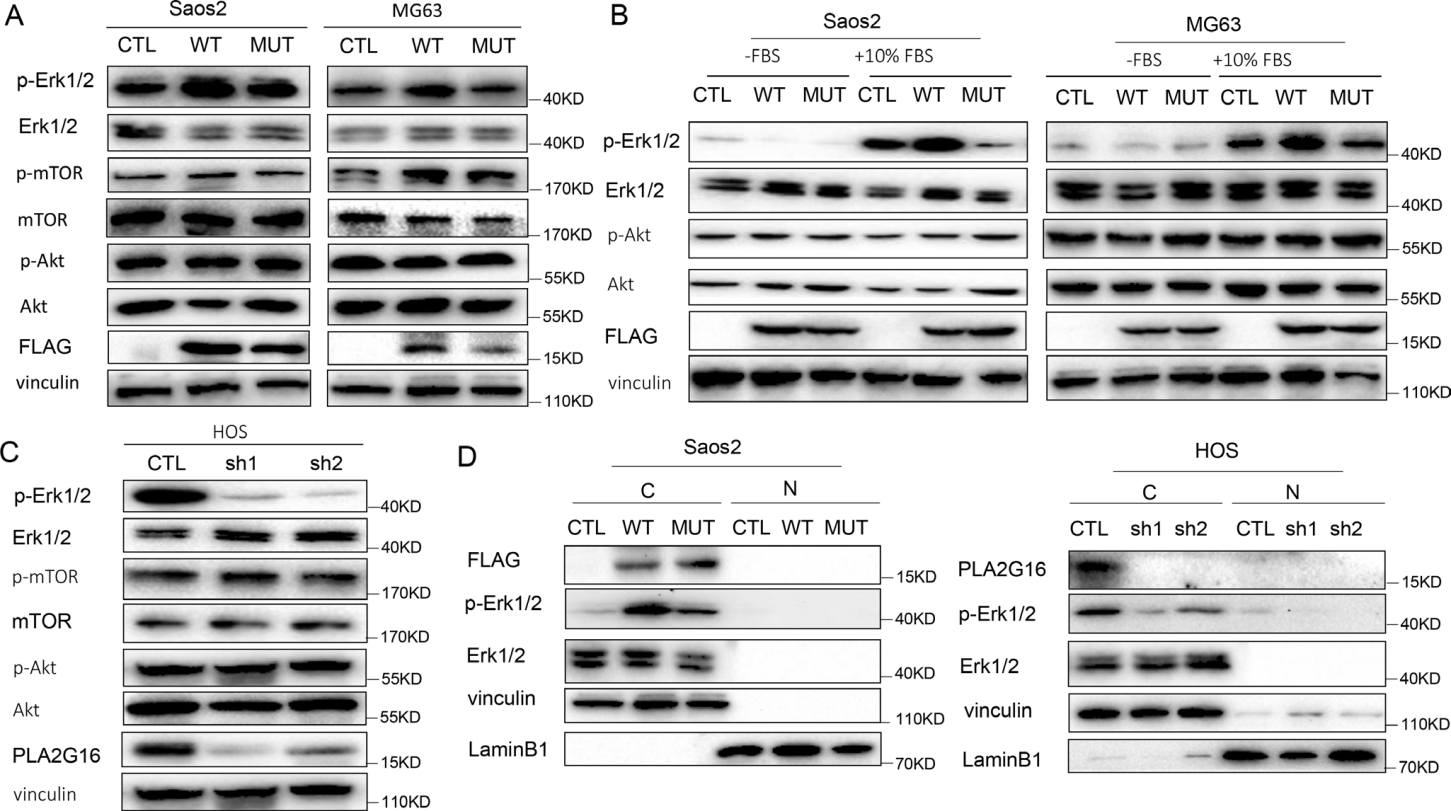
PLA2G16 localizes to the cytoplasm and induces the phosphorylation of ERK1/2 (**A**) PLA2G16 overexpression increased ERK1/2 phosphorylation in Saos2 and MG63 cells with the overexpression of PLA2G16. (**B**) PLA2G16 increased ERK phosphorylation after stimulation with FBS for 5 min. Cells were starved for 12 hours (−FBS) and then stimulated with 10% FBS (+10% FBS). (**C**) PLA2G16 knockdown decreased ERK1/2 phosphorylation in HOS cells. Vinculin was used as the loading control. (**D**) Cytoplasmic localization of PLA2G16 in Saos2 and HOS cell. Cytoplasm and nuclear protein were extracted respectively and subjected to western blotting analysis. Vinculin was used as cytoplasmic loading control and LaminB1 was used as nuclear loading control. C and N indicate cytoplasm and nuclear, respectively.

It is well known that phospholipases modulate multiple downstream pathways in cancer cells [32]. To identify the pathways through which PLA2G16 increased the oncogenic phenotype of osteosarcoma cell we tested the activities of several candidate downstream pathways by western blot analyses. *PLA2G16* overexpression clearly increased the phosphorylation of ERK1/2 both at the normal culture condition (Figure 6A) and fetal bovine serum stimulation after overnight starvation (Figure 6B) in both Saos2 and MG63 cells, suggesting activation of the MAPK pathway is downstream of PLA2G16. Consistently, ERK1/2 phosphorylation was markedly decreased not only in *PLA2G16* knockdown HOS cells (Figure 6C) but also in LM7 cells (Figure 4S).

Previously, the function of PLA2G16 in tumor development was reported to be associated with its intracellular localization. Nuclear localized PLA2G16 has been shown to have tumor suppressive activity whereas a cytoplasmic localized PLA2G12 has oncogenic effects [27]. Therefore, we extracted the cytoplasm and nuclear fractions from Saos2 and HOS cell respectively to determine localization of PLA2G16. Interestingly, we found that PLA2G16 was largely cytoplasmic and associated with higher phosphorylation of ERK1/2 (Figure 6D), consistently with the study in NSCLC [27].

**Figure 7:**
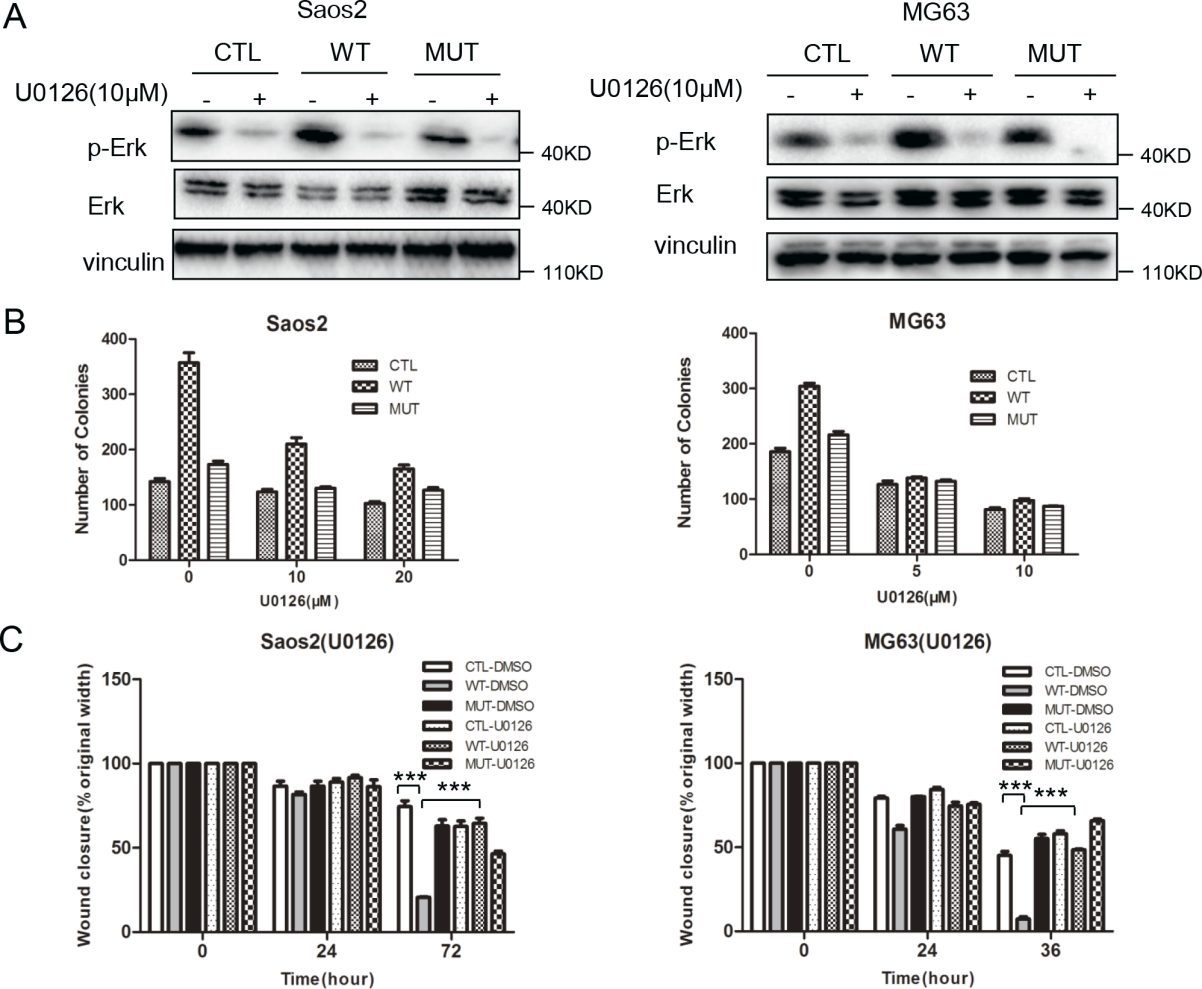
Inhibition of the MAPK pathway suppresses low density colony formation and migration in osteosarcoma cells (**A**) U0126 significantly inhibits Erk1/2 phosphorylation in Saos2 and MG63 cells. Cells were incubated with 10 μM U0126 or DMSO before the proteins were harvested and subjected to western blotting. The induction of low density colony formation (**B**) and cell migration (**C**) mediated by PLA2G16 overexpression was inhibited by U0126. In low density colony formation assay, one thousand cells were plated to 6-well plates and incubated with normal medium containing DMSO (CTL) or U0126 for 14 days before colonies were stained and counted. In wound healing assay, cells were incubated with DMSO or 10 μM U0126 for 24 hours. One way ANOVA was used for statistical analyses to compare between PLA2G16 overexpression (WT) and empty vector pBABE-puro (CTL) cells ** indicates *p* < 0.01.

To determine the functional relevance of MAPK pathway activation mediated by PLA2G16 expression in osteosarcoma progression, we used U0126 monoethanolate (a MEK specific inhibitor) to inhibit the phosphorylation of ERK1/2 (Figure 7A) and examined the clonogenic survival and migration abilities in Saos2 and MG63 cells. The low-density colony formation assays showed that the colongenicity induced by PLA2G16 overexpression was specifically inhibited by U0126 both in Saos2 (from 2.15 fold induction reduced to 1.70 fold under 10 μM and 1.60 under 20 μM) and MG63 cells (from 1.64 fold induction reduced to 1.06 fold under 5 μM and 1.19 fold under 10 μM) (Figure 7B and Figure S5A). Wound-healing assays showed migration was similar among PLA2G16 (WT), PLA2G16-C113 (MUT) or the control vector (CTL) cell lines in the presence of U0126 (Figure 7C and Figure S5B), indicating U0126 also specifically inhibited the migration induced by PLA2G16 overexpression. Thus, our data demonstrated that PLA2G16 may function as an oncogene in part by activating MAPK pathway.

### PLA2G16 contributes tumorigenesis *in vivo*

**Figure 8:**
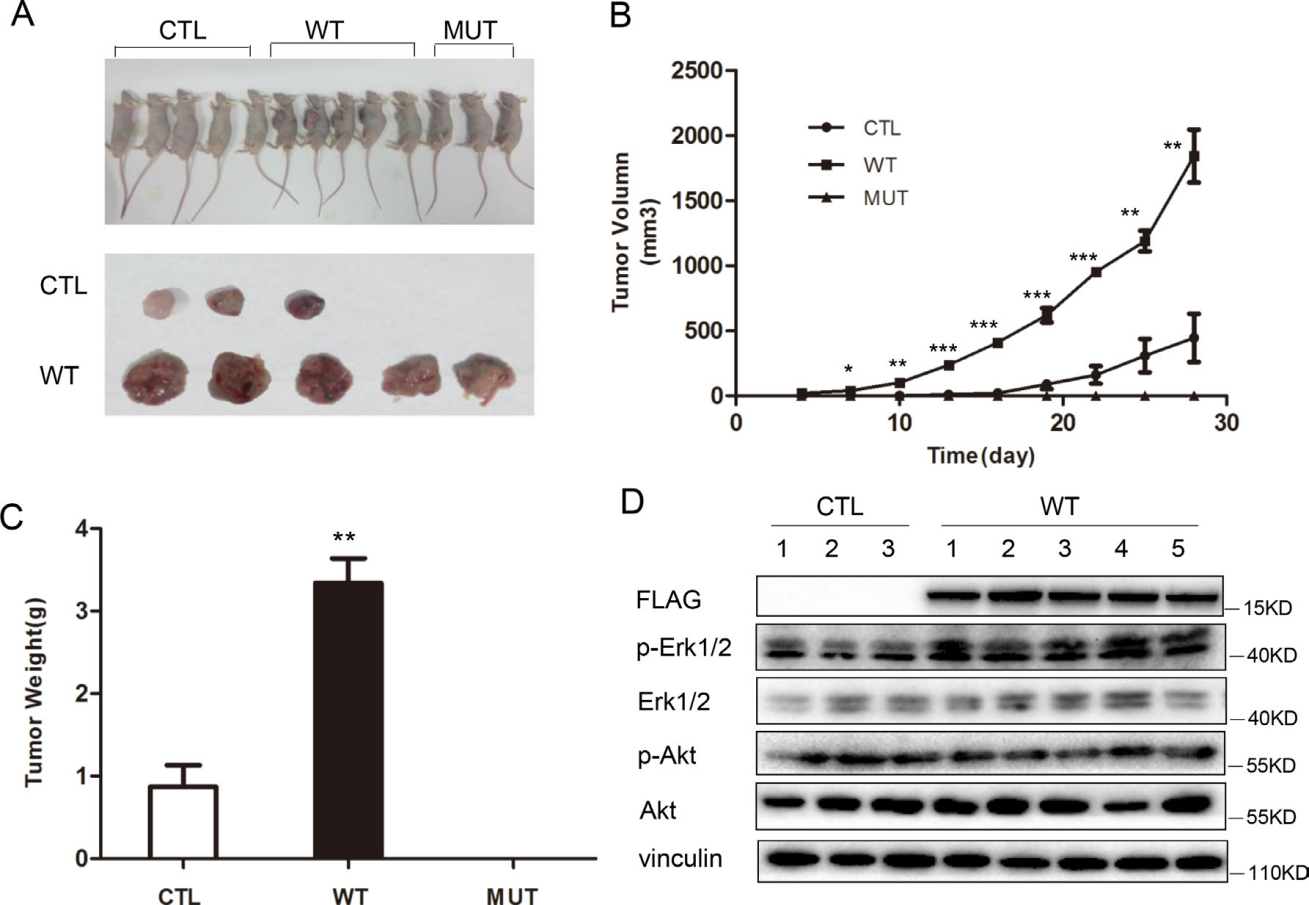
PLA2G16 promotes Saos2 cell tumorigenesis *in vivo* (**A**) Tumor bearing nude mice from the xenograft experiments using *PLA2G16* or *PLA2G16-C113S* overexpressing Saos2 cells. (**B**) Tumor growth curve plotted using the mean tumor volumes. (**C**) Average tumor weight was determined after nude mice were sacrificed and tumors excised. (**D**) Western blot analyses from the tumor protein extracts. Results are shown as the mean ± SD and One way ANOVA was used for statistical analyses, * indicates *p* < 0.5, ** indicates *p* < 0.01, *** indicates *p* < 0.001.

To determine the effect of PLA2G16 overexpression in osteosarcoma cells *in vivo*, we injected our Saos2 cell line panel subcutaneously into Balb/c nude mice. After tumors were visible (about 2 week after injection), the length and width were measured and the volume was calculated every 3 days. Only three out of five mice developed tumors from the control group, and the mice injected with PLA2G16-C113 mutant expressing cells did not develop tumors (3 out of 3), All five mice injected with PLA2G16 overexpressing cells developed tumors (Figure 8A). The tumors grew much faster in the mice with PLA2G16 overexpressing cells (Figure 8B), and the average tumor volumes were significantly larger than the CTL group (Figure 8A, 8C). Western blots performed from tumor lysates showed that PLA2G16 overexpression group (WT) is associated higher ERK1/2 phosphorylation (Figure 8D), further supporting that activation of ERK1/2 phosphorylation in tumors is downstream of PLA2G16.

### PLA2G16 expression is associated with ERK1/2 phosphorylation in osteosarcoma patients and predicts for poor overall and metastasis-free survival

To examine the relevance of the PLA2G16 and ERK1/2 phosphorylation in primary patient samples, an osteosarcoma tissue array was generated including thirty-five (39.8%) female and 53 (60.2%) male patients with histologically confirmed osteosarcoma. The median age of the patients was 19 years, with an age range from 1 to 72 years at the time of surgery. At the end of the last follow up, 41 patients died, primarily due to metastases (30/41, 73%). The clinical and histopathologic details of the 88 cases are listed in Tables 1 and 2. Positive staining of PLA2G16 and p-ERK1/2 are mainly present on the cytoplasm of the tumor cells (Figure 9A), which is consistent with the western blot analyses from PLA2G16 overexpression cells (Figure 6D). To elucidate the biologic significance, we investigated the association of clinicopathologic features and PLA2G16 and p-ERK1/2 expression levels. As shown in Table 1, PLA2G16 and p-ERK1/2 were associated with lung metastasis (both *P* < 0.05), but not with gender, age at diagnosis, tumor location or histological classification (*P* > 0.05). The 3-and 5-year OS and MFS rates were 75.0% and 62.6%, 65.5% and 57.7%, respectively, for the entire study population. PLA2G16 was an independent prognostic factor for OS and MFS again for this study group (Figure 9B). The PLA2G16 positive group had significantly lower 5-year OS (55.1% vs 71.7%; *P* = 0.05) and MFS rates (47.9% vs 69.4%; *P* = 0.036) than that of the remaining cases, respectively (Table 2). Interestingly, p-ERK1/2 was also a prognostic factor for both OS and MFS (Figure 9C). The p-ERK1/2 negative group had significantly greater 5-year OS (80.5% vs 53.0%, *P* = 0.009) and MFS (76.8% vs 46.9%, *P* = 0.011) rates than those of the p-ERK1/2 positive group. PLA2G16 and p-ERK1/2 expression was positive in 48 (54.5%) and 57 (64.8%) cases, respectively. There was a significant correlation between the expression of PLA2G16 and p-ERK1/2 (*P* = 0.002) in these osteosarcoma samples (Table 3). Furthermore, when PLA2G16 and p-ERK1/2 expression were both taken into consideration, 21 patients (23.9%) were both PLA2G16 (PLA2G16-) and p-ERK1/2 negative (p-ERK1/2-), and 38 patients (43.2%) were PLA2G16 (PLA2G16+) and p-ERK1/2 positive (p-ERK1/2+) simultaneously. There were 29 cases (32.9%) either PLA2G16+ or p-ERK1/2+ positive. The prognosis of PLA2G16 and p-ERK1/2 double negative group was the best with 5-year OS and MFS at 85.4% and 85.7%, respectively; *P* = 0.020, *P* = 0.016) (Figure 9D). The 5-year OS (61.5%) and MFS (54.1%) rates for the group of PLA2G16-p-ERK1/2+ or PLA2G16+p-ERK1/2-dropped dramatically (Table 2, both *P* < 0.05 and Figure 9D), yet 5-year OS (51.1%) and MFS (44.7%) rates in the group for double positive for PLA2G16 and p-ERK1/2 were further decreased (Figure 9D), indicating that PLA2G16 high expression and p-ERK1/2 may be additive effect for prognosis. To determine the predictive power of PLA2G16 and p-ERK1/2 protein expression for metastasis, we calculated the area under the Receiver Operating Characteristic curve (ROC) for the following three prognostic models (Table 4): PLA2G16 expression (+ vs.−), p-ERK1/2 expression (+ vs. −), PLA2G16 combined with p-ERK1/2 expression (both + vs. others). In the present study, PLA2G16 and p-ERK1/2 positive rates (75.8% vs 41.8%, *P* = 0.002; 81.8% vs 54.5%, *P* = 0.010; respectively; Table 1) in patients with lung metastasis were significantly higher than those non-metastatic ones. Both PLA2G16 and p-ERK1/2 could be potential biomarkers for predictive osteosarcoma lung metastasis and yielded an area under the ROC curve (AUC) of 0.670 and 0.636, respectively. Combined ROC analyses revealed an AUC of 0.706, indicating the additive effect in the predictive value of both genes (Table 4). These data strongly support that PLA2G16 contributes osteosarcoma metastasis and it may be mediated through MAPK pathway activation in patients.

**Table 1:** Relationship between PLA2G16 and p-ERK1/2 and clinicopathologic factors of patients

Variables	Total	PLA2G16 expression (*n*)			p-ERK1/2 expression (*n*)		
	(*n* = 88)	Positive	Negative	*P*-values	Positive	Negative	*P*-values
**Gender**							
Male	53 (60.2%)	29	24	0.968	32	21	0.288
Female	35 (39.8%)	19	16		25	10	
**Age at diagnosis**							
≤ 20 years	54 (61.4%)	31	23	0.497	34	20	0.654
> 20 years	34 (38.6%)	17	17		23	11	
**Tumor location**							
Femur	45 (51.1%)	22	23	0.111	29	17	0.686
Tibia	16 (18.2%)	8	8		9	7	
Humerus	10 (11.4%)	8	2		7	3	
Fibula	7 (7.9%)	6	1		6	1	
Others	10 (11.4%)	4	6		6	4	
**Histological classification**							
Osteoblastic	60 (68.2%)	34	26	0.628	41	19	0.588
Chondroblastic	16 (18.2%)	9	7		9	7	
Others	12 (13.6%)	5	7		7	5	
**Lung metastasis**							
Yes	33 (37.5%)	25	8	0.002	27	6	0.010
No	55 (62.5%)	23	32		30	25	

**Table 2:** Clinicopathologic patient characteristics and univariate survival analysis

Variables	Patients (*n* = 88)	5-y OS rate	*P*-values	5-y MFS rate	*P*-values
**Gender**					
Male	53	61.3%	0.953	57.0%	0.819
Female	35	64.8%		58.6%	
**Age at diagnosis**					
≤ 20 years	54	59.3%	0.648	57.6%	0.689
> 20 years	34	67.2%		58.5%	
**Tumor location**					
Femur	45	68.2%	0.318	63.3%	0.342
Tibia	16	48.2%		50.0%	
Humerus	10	53.3%		58.3%	
Fibula	7	42.9%		28.6%	
Others	10	80.0%		68.6%	
**Histological classification**					
Osteoblastic	60	59.9%	0.669	56.8%	0.702
Chondroblastic	16	56.3%		56.3%	
Others	12	83.3%		64.2%	
**Lung metastasis**					
Yes	33	21.2%	*P* < 0.001	4.0%	*P* < 0.001
No	55	87.1%		87.1%	
**PLA2G16**					
Negative	40	71.7%	0.050	69.4%	0.036
Positive	48	55.1%		47.9%	
**p-ERK1/2**					
Negative	31	80.5%	0.009	76.8%	0.011
Positive	57	53.0%		46.9%	
**PLA2G16 plus p-ERK1/2**					
PLA2G16-, p-ERK1/2−	21	85.4%	0.020	85.7%	0.016
PLA2G16-, p-ERK1/2 + and PLA2G16+, p-ERK1/2−	29	61.5%		54.1%	
PLA2G16+, p-ERK1/2+	38	51.1%		44.7%	

**Table 3:** The correlation between the expression of p-ERK1/2 and PLA2G16

p-ERK1/2 expression	PLA2G16 expression	
	Negative	Positive
Negative	21 (52.5%)	10 (20.8%)
Positive	19 (47.5%)	38 (79.2%)
Total	40	48
*P* value	0.002	

**Table 4:** The predictive value of PLA2G16 and p-ERK1/2 for lung metastasis

Variables	Lung metastasis		Total	Area under ROC	95% CI	*P* values
	**Yes**	**No**				
PLA2G16+	25	23	48	0.670	0.554–0.786	0.008
PLA2G16−	8	32	40			
p-ERK1/2+	27	30	57	0.636	0.519–0.754	0.033
p-ERK1/2−	6	25	31			
PLA2G16+, p-ERK1/2+	21	17	38	0.706	0.597–0.814	0.001
other#	12	38	50			

# Including PLA2G16+, p-ERK1/2−; PLA2G16-, p-ERK1/2+; and PLA2G16−, p-ERK1/2−.

**Figure 9:**
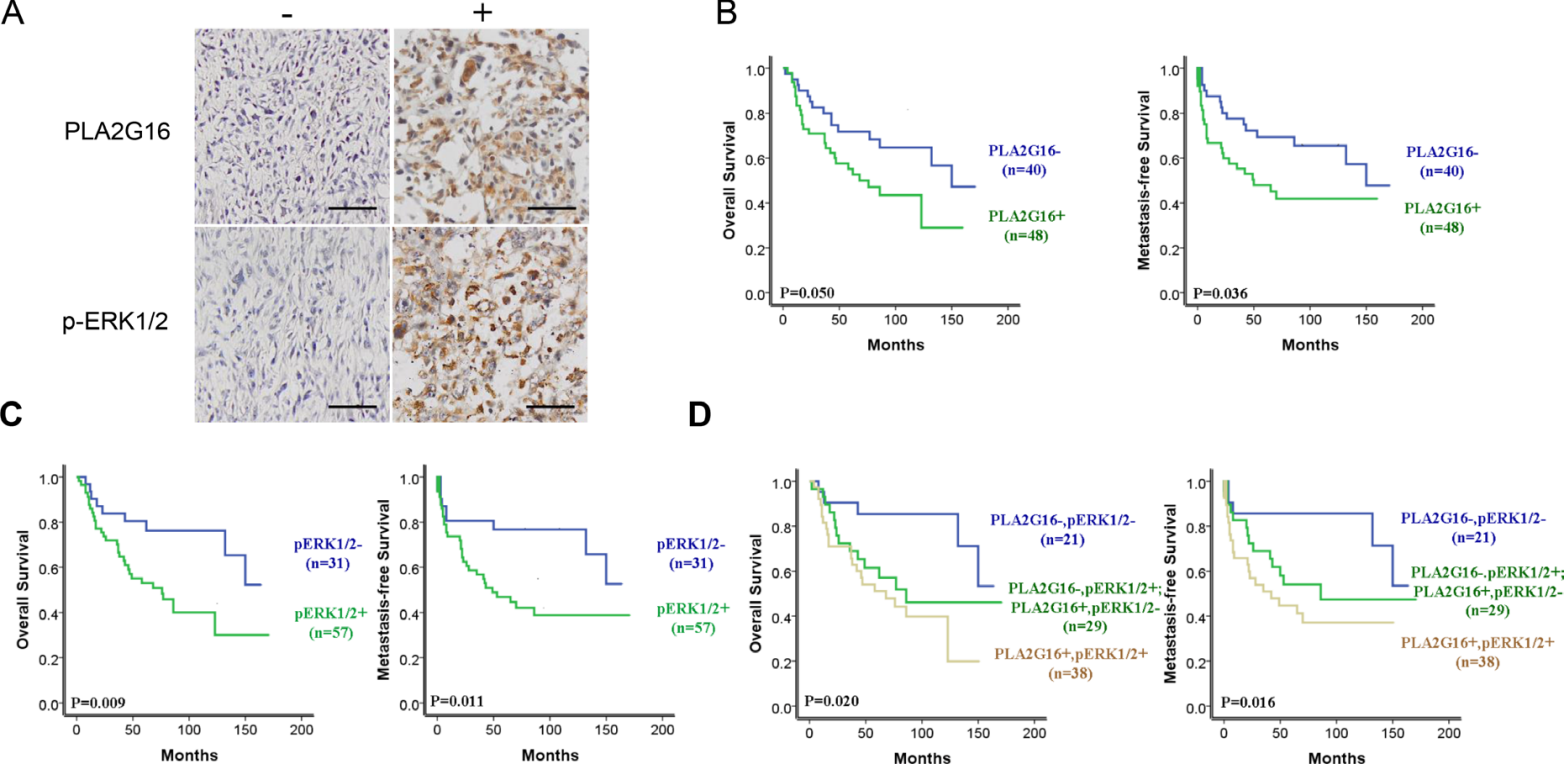
The phosphorylation of ERK1/2 and PLA2G16 overexpression are associated with poor prognosis and metastasis in osteosarcoma patients (**A**) Representative images showing the positive immunohistochemical staining for PLA2G16 and p-ERK1/2 in the tissue microarray at magnification of 400 (−) indicated as negative and (+) indicated as positive staining, scale bar = 100 μm. Positive PLA2G16 (**B**) and p-ERK1/2 (**C**) immunohistochemical staining was associated with the worse overall survival (OS) and metastasis-free survival (MFS) determined by Kaplan-Meier analysis. (**D**) The additive effect of PLA2G16 and p-ERK1/2 staining in predicting overall survival and metastasis-free survival in osteosarcoma patients determined by Kaplan-Meier analysis.

## DISCUSSION

PLA2G16 was originally called a class II tumor suppressor based on studies that demonstrated an inhibitory role on HRAS. Recent recent studies have demonstrated that HRAS expression is sometimes a good prognostic factor in various cancer types, including breast cancer and neuroblastoma at the early stage [33, 34]. Thus, the inhibitory role of PLA2G16 on HRAS in tumor progression and metastasis is very likely dependent on the tumor types and tumor stages [35]. PLA2G16 can also inhibit well-known tumor suppressor PP2A activity, suggesting that PLA2G16 may have oncogenic activity in some tumors. Additionally, PLA2G16 is now known to produce LPA and FFA, and multiple signal pathways are directly or indirectly regulated by LPA and FFA in tumors. Taken together, these results suggest that PLA2G16 has different roles in different cell contexts. Our studies indicate that PLA2G16 overexpression is associated with osteosarcoma progression and can be a potential biomarker to predict osteosarcoma lung metastasis [28, 29]. Herein we further demonstrate that PLA2G16 expression promotes migration, invasion, clonogenic survival, and anchorage independent growth of human osteosarcoma cell lines. Mechanistically we show that PLA2G16 actives the MAPK pathway to promote tumor progression *in vitro* and *in vivo*. Additionally, analysis of a human osteosarcoma tissue microarray indicates a strong correlation between PLA2G16 expression and activation of the MAPK pathway. An additive effect of PLA2G16 expression and ERK1/2 phosphorylation is also shown to predict patient survival and metastasis. Thus, our data strongly support an oncogenic role for *PLA2G16* in promoting osteosarcoma progression and metastasis.

We also demonstrate that PLA2G16 overexpression reduced sensitivity to chemotherapeutic drugs in both a dose and time-dependent manner, suggesting the development of small molecular inhibitors against PLA2G16 could be used to increase the sensitivity of osteosarcoma standard chemotherapy. The mechanisms of PLA2G16 activity in altered drug sensitivity is still need to be investigated in the future.

The main cause of mortality in osteosarcoma patients is metastasis. Patients with localized tumors have a 5-year survival rate in the range of 60% to 80%. In contrast, patients with metastasis have a 5-year survival rate between 15% to 30% [1, 36]. Additionally, osteosarcoma is generally characterized by highly complex karyotypes and unknown origin [37]. Thus, it has been difficult to identify osteosarcoma-specific therapeutic targets. Based on the data in this study, we believe that PLA2G16 is a good therapeutic target for osteosarcoma patients. Additionally, the overexpression of a mutant PLA2G16 construct in osteosarcoma cells appears to have dominant-negative activity over the wild-type enzyme as the endogenous levels of p-Erk are reduced (Figure 6) and the xenograft model also shows reduced tumor growth in cells expressing the mutant PLA2G16 compared to empty vector controls (Figure 8B, 8C). These data further suggest that an inhibitor against PLA2G16 may have profound effects in the treatment of osteosarcoma and would suppress tumor growth by inhibiting the MAPK pathway. It will be interesting to examine the effect of inhibition of PLA2G16 phospholipase activity and inactivating the MAPK pathway as a combination therapy in the future. Combined, our data support the development of a specific PLA2G16 inhibitor or alternative strategies, such as *in vivo* gene targeting of PLA2G16 by CRISPR/Cas9 technology, for the treatment of osteosarcomas.

## MATERIALS AND METHODS

### Cell lines and culture

Osteosarcoma cells (Saos2, MG63 and HOS) were obtained from China Infrastructure of Cell Line Resource and cultured in DMEM medium with 10% fetal bovine serum and 100 U/ml penicillin and 100 U/ml streptomycin.

Retrovirus or lentivirus were produced using 293T cells and were collected twice between 36 h and 72 h to infect osteosarcoma cells followed by manufacturer's instructions. After infection, cells were selected by puromycin for 2 weeks and the expression level of PLA2G16 was confirmed by qRT-PCR and western blot.

### Quantitative RT-PCR analysis and western blots

Total mRNA was extracted with Trizol reagent (Invitrogen, Carlsbad, CA, USA). RNA was reverse transcribed to cDNA with FastQuant RT Kit (with gDNase) (Tiangen Biotech, China) according to the manufacturer's instructions. For quantitative RT-PCR system, SYBR Premix Taq (Tiangen Biotech, China) was used. *PLA2G16* gene-specific primers were previously described [28]. *GAPDH* genes was used as internal control.

Cells were lysed with RIPA lysis buffer and protein were resolved by SDS-PAGE and transferred to PVDF membrane. The antibodies including vinculin (Santa Cruz, CA), pAkt, Akt, p-Erk, Erk, p-mTOR, m-TOR (Cell Signaling Technology, MA) were incubated at the dilution specified by manufacturer at 4°C overnight.

### Cell proliferation with or without anticancer drugs

Cell proliferation was assessed by 3-(4, 5-dimethyl-2-thiazolyl)-2, 5-diphenyl-2-H-tetrazolium bromide (MTT) assay. All stable cell lines were seeded into 96-well plates at 1 × 10^3^/well. For cell proliferation assay, 0.5 mg/ml MTT solution was added to each well for four hours and measured at the absorbance of 570 nm by multifunctional micro plate reader.

### Colony formation (clonogenicity) and soft agar assays

Stable transfected cells were seeded in triplicate into 6-well plates. Culture medium was changed every 3 days and cells were grown for 10–14 days until colonies were formed. Colonies were fixed with paraformaldehyde and stained with crystal violet for 15 minutes at room temperature. Colonies that contained more than 50 cells were counted.

The anchorage-independent growth was determined by soft agar colony formation assay. Cells (1 × 10^3^) were suspended in 1 ml of 0.35% agar supplemented with 10% FBS and DMEM medium and plated on a 0.7% agar coated 6-well plate. The plates were incubated for 2–3 weeks and then stained with 0.005% crystal violet and the number of colonies were counted.

### Migration and invasion assay

Cell migration was determined by wound-healing assay. Cancer cells were seeded at a concentration of 3 × 10^5^ cells/ml into the plates with a culture insert (Inbidi, Madison, Wisconsin) to form a gap of 500 μm according to the manufacturer's instruction. After removing the insert, cells that migrated into the wound area were photographed by inverted microscope. The wound closure width is calculated as following: (12– to 72-hour width/0–hourwidth)*100%.

Matrigel Transwells (BD Biosciences, NJ) were used for *in vitro* invasion assay. Briefly, the insert standing in 24-well culture plate were coated with 50 ul Matrigel in serum-free DMEM medium and dried overnight. 1 × 10^5^ cells in 100 μl serum-free medium were added to the insert. The lower chamber was added 500 μl DMEM medium containing 10% FBS. Cells were incubated for 36 hours and the cells that penetrated the membrane were counted after fixing with the 4% paraformaldehyde and staining with 1% crystal violet.

### Animal experiments

Four-week-old male nude mice (Balb/c, nu/nu; SPF laboratory animal center of Dalian Medical University, Dalian, China) were housed under pathogen-free condition. All animal experiments were approved by the Animal Ethics Committee of Dalian Medical University. Three millions Saos2 cells (at the concentration of 3 × 10^7^ cells/ml) with overexpression of wild type or mutant PLA2G16 were suspended in 50% Matrigel and subcutaneously injected into nude mice. Tumor size was calculated by ½ a^2^b (a is the short axis and b is the long axis of tumor). Growth curve were plotted with the mean tumor volume ± SEM. Thirty days after injection, the animals were sacrificed and tumor was harvested, measured, weighed and fixed in 10% formalin. Tumor weight of each animal was calculated as mean ± SD from animal in each group. Protein lysate were prepared from the tumors for western blot analysis.

### Tissue microarray and immunohistochemistry

TMA construction was previously described in detail [29]. Immunohistochemistry was performed with PLA2G16 antibody at a 1:100 dilution (Item No. 10337, Cayman Chemical, Ann Arbor, USA) and p-ERK1/2 monoclonal antibody at a 1:300 dilution (#4370, Cell Signaling Technology, Boston, USA).

### Statistical analysis

Correlations between different immunoreactivity and clinical variables were assessed using Pearson's chi-squared test or Fisher's exact test. The prognostic effects of PLA2G16 and p-ERK1/2 were evaluated using the Kaplan–Meier method and compared using the log-rank test. The Spearman rank test was applied to demonstrate correlations. Receiver-operating characteristics (ROC) curves were established to evaluate the predictive value of PLA2G16 and p-ERK1/2 expression for metastasis from primary osteosarcoma, a larger area under the ROC corresponded to greater prognostic potential for a specific factor. Overall survival (OS) was defined as the interval between date of diagnosis and death or the last observation. For the metastasis free survival (MFS) analysis, the duration was defined as the time from diagnosis until the occurrence of metastasis or the last follow up. If these patients had metastatic disease at diagnosis, the event was considered time 0. For all the analyses, a two-sided *P* value ≤ 0.05 was considered statistically significant. All statistical analyses were performed using the SPSS for windows, version 18.0 (SPSS, Inc, Chicago, IL).

## SUPPLEMENTARY MATERIALS FIGURES


