# Supplementary material for: PLA2G16 promotes osteosarcoma metastasis and drug resistance via the MAPK pathway
Source: Oncotarget. 2016 Feb 25;7(14):18021–35. doi: 10.18632/oncotarget.7694 (PMC4951268; doi:10.18632/oncotarget.7694)
Supplement: Supplementary file 1 [file oncotarget-07-18021-s001.pdf]

PLA2G16 promotes osteosarcoma metastasis and drug resistance via MAPK pathway

Supplementary Materials

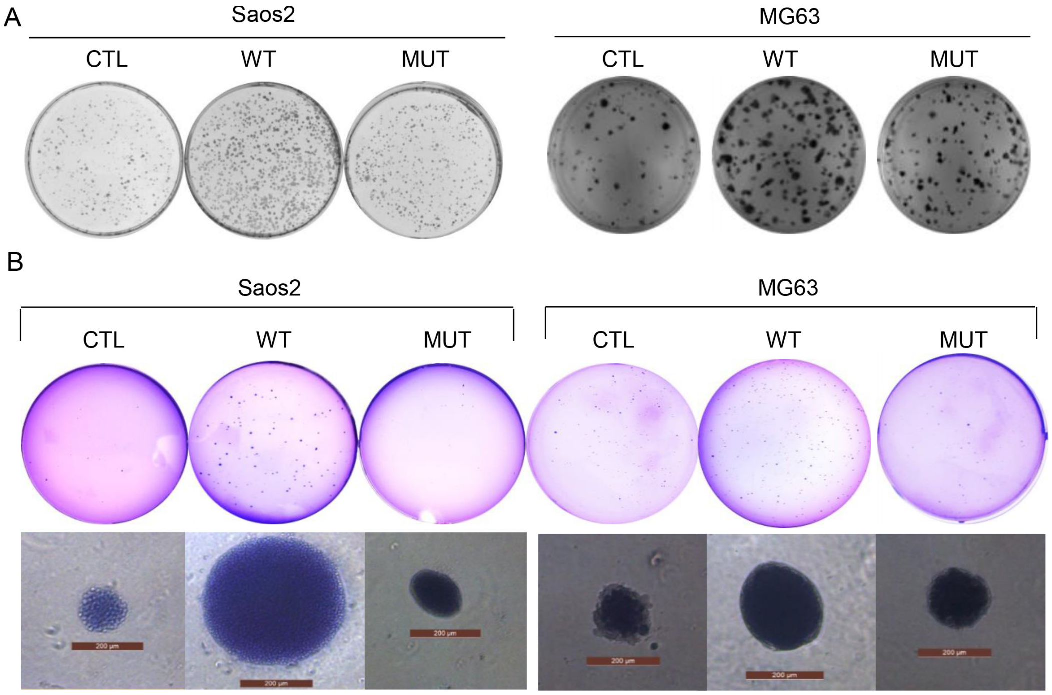

Supplementary Figure S1: PLA2G16 overexpression significantly increases clonogenicity and anchorage-independent of growth in osteosarcoma cell lines. (**A**) Low magnification photos of colony formation assays using Saos2 and MG63 cells with PLA2G16 overexpression. (**B**) Soft agar assays of PLA2G16 overexpression cells under low (top) and high magnification (bottom).

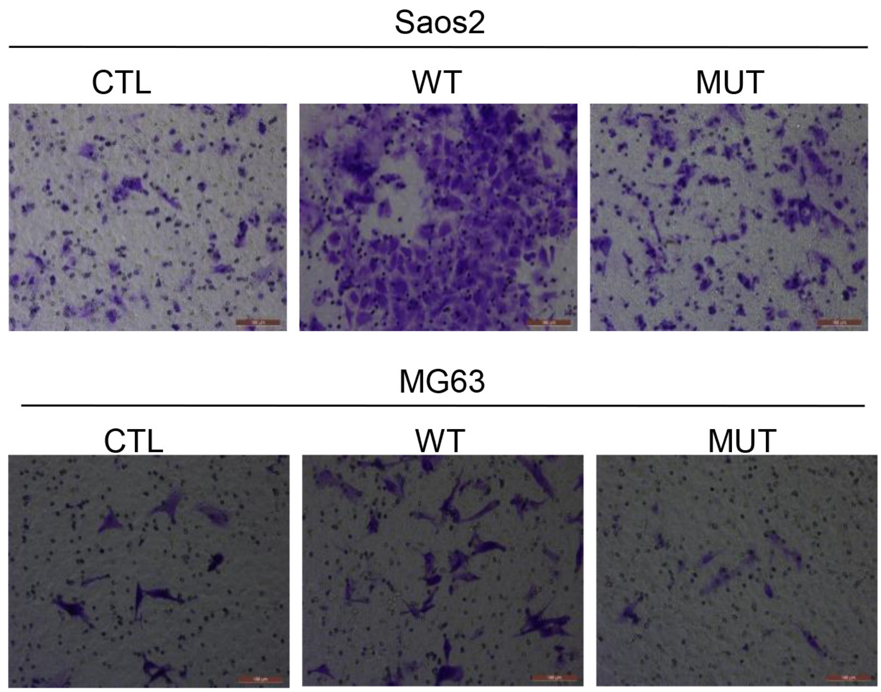

Supplementary Figure S2: Overexpression of PLA2G16 increases cell invasion by matrigel transwell assay. All photos were taken by inverted microscope at a 100X magnification. CTL, p-BABE-puro; WT, pBABE-FLAG-PLA2G16; MUT, pBABE-FLAG-PLA2G16-C113S.

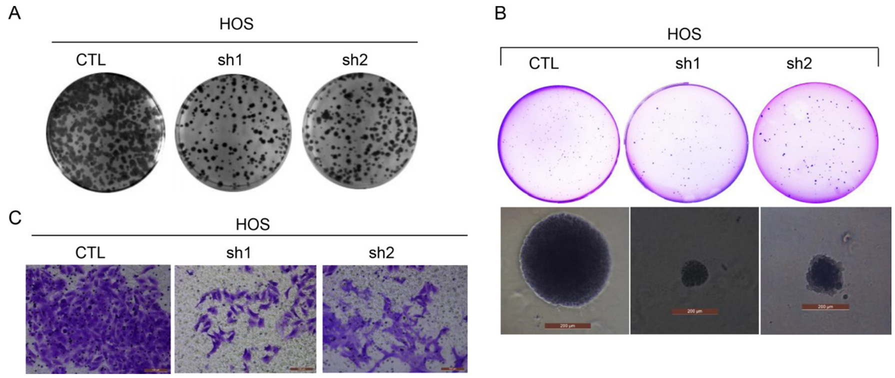

Supplementary Figure S3: PLA2G16 knockdown reduces clonogenicity, anchorage-independent growth and invasion in HOS cells. (**A**) Low magnification photos of colony formation assay (**B**) Soft agar assay under low (top) and high magnification (bottom). (**C**) Knockdown of PLA2G16 decreases cell invasion.

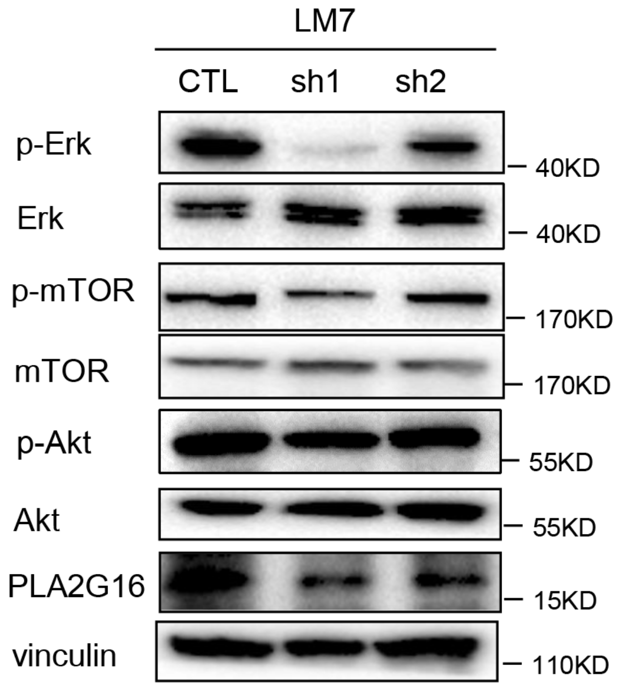

Supplementary Figure S4: PLA2G16 knockdown decreases Erk1/2 phosphorylation in LM7 cells. Cells were incubated in normal culture condition before cell lysis were subjected to immunoblotting with indicated antibodies. Vinculin was used as the loading control.

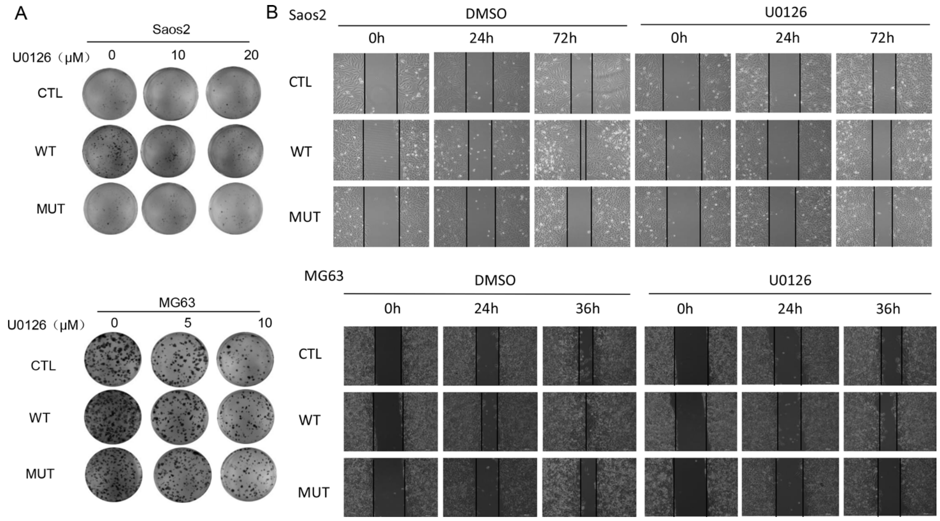

Supplementary Figure S5: Inhibition of MAPK pathway represses PLA2G16 mediated clonogenicity and migration. Low density colony formation assays (**A**) and Wound-healing assays (**B**) were performed in U0126 treated Saos2 and MG63 cells. Photos were taken at low magnification or under inverted microscope respectively. CTL, p-BABE-puro; WT, pBABE-FLAG-PLA2G16; MUT, pBABE-FLAG- PLA2G16-C113S.
